# Comparison between Canadian Canola Harvest and Export Surveys

**DOI:** 10.3390/plants5030030

**Published:** 2016-07-20

**Authors:** Véronique J. Barthet

**Affiliations:** Grain Research Laboratory, Canadian Grain Commission, 1404-303 Main St., Winnipeg, MB R3C 3G8, Canada; veronique.barthet@grainscanada.gc.ca; Tel.: +1-204-984-5174

**Keywords:** canola, Canadian export quality, harvest survey, oil, protein, chlorophyll, glucosinolates, oleic acid, α-linolenic acid, iodine value, free fatty acid

## Abstract

Parameters, such as oil, protein, glucosinolates, chlorophyll content and fatty acid composition, were determined using reference methods for both harvest survey samples and Canadian Canola exports. Canola harvest survey and export data were assessed to evaluate if canola harvest survey data can be extrapolated to predict the quality of the Canadian canola exports. There were some differences in some measured parameters between harvest and export data, while other parameters showed little difference. Protein content and fatty acid composition showed very similar data for harvest and export averages. Canadian export data showed lower oil content when compared to the oil content of harvest survey was mainly due to a diluting effect of dockage in the export cargoes which remained constant over the years (1.7% to 1.9%). Chlorophyll was the least predictable parameter; dockage quality as well as commingling of the other grades in Canola No. 1 Canada affected the chlorophyll content of the exports. Free fatty acids (FFA) were also different for the export and harvest survey. FFA levels are affected by storage conditions; they increase during the shipping season and, therefore, are difficult to predict from their harvest survey averages.

## 1. Introduction

Canola production steadily increased in Canada to reach over 17 million tonnes in 2013. In 2014, the Canola Council of Canada released a new strategy (“keep it coming”) to encourage the Canadian canola industry to increase canola production to 26 million tonnes and to acquire new markets for the increased production. On a yearly average, over half of the seed production is exported to China, Japan, Mexico and the US, which are the main markets in Canada.

In Canada, canola is sold based on grades defined by the Canadian grain guide [1]. These grades have been established to allow for rapid segregation upon delivery at the various points of the grain supply chain (primary or terminal elevator, feed mill or crushing plant). While the Canadian grain guide defines the grades of Canadian canola, there are also other important chemical quality parameters for canola seed. These include oil, protein, chlorophyll, glucosinolates, free fatty acids and the fatty acid composition. Oil is an important quality factor since canola, being an oilseed, is processed to produce high quality oil. Protein content is important in order to produce good quality feed for animals, especially dairy cows. Chlorophyll content is a negative quality factor for Canadian canola. The short growing season in Canada is usually responsible for seed containing non-negligible chlorophyll amounts. This chlorophyll is found in crude oil and has to be removed from the oil during refining to produce clear canola oil for commercial use.

Genetic and environmental growing conditions affect canola quality parameters. For example, there is an inverse relationship with the oil content of the seed and temperature; hot growing conditions will lead to lower oil content when compared to cooler conditions. The opposite effect is found for the protein content of the seeds. Canola chlorophyll content is greatly affected by the growing conditions; a longer growing season will give a low chlorophyll content whereas a shorter season, often due to early frost, will give seeds with very high chlorophyll content

This paper deals with a comparison of the data obtained from the yearly harvest survey program (Harvest Sample Data or HSD) of the Canadian Grain Commission and from the export monitoring program (Export Cargo Data or ECD) also conducted by the Canadian Grain Commission on officially inspected cargoes.

## 2. Results and Discussion

### 2.1. Harvest Survey Data

In 1927, the Canadian Grain Commission started a voluntary survey for wheat in the western provinces of Canada. In 1956, rapeseed (canola) was included in the annual harvest survey. Every year, samples of canola from western Canada are sent by producers, grain elevators and processors to the Canadian Grain Commission where the samples are graded and analyzed to produce annual reports describing the quality of the harvest of western Canadian grains. For this paper, only canola harvest sample data (HSD) reported for the western provinces (Manitoba, Saskatchewan, Alberta and the Peace River area of British Columbia) from 2000 to 2014 are used (Table 1).

**Figure 1:**
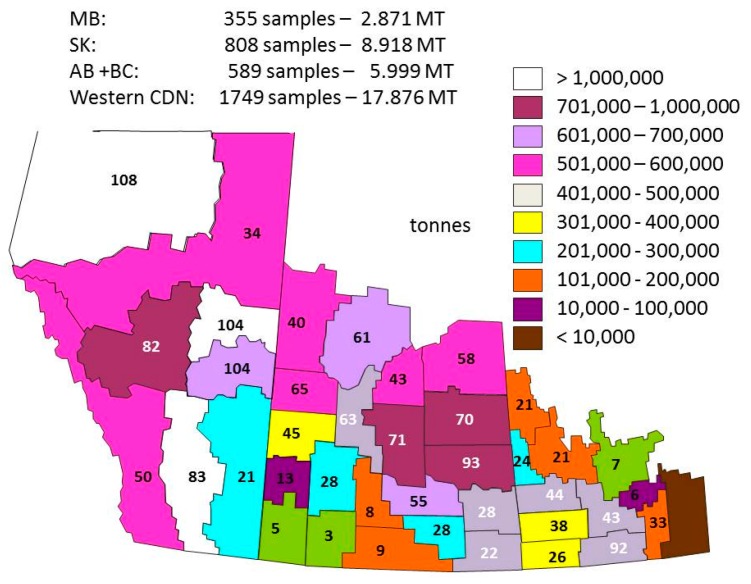
Scheme of the western provinces (Manitoba, Saskatchewan and Alberta) showing each crop district present in the 2013 production survey and the number of samples received for the harvest survey in 2014 for each crop district.

The averages for each of the provinces for the harvest survey are calculated using crop district data (Figure 1). HSD for each crop district are weighted by production values and by the number of samples in the grade. These in turn are used to calculate the provincial averages.

Figure 1 shows an example of crop district production and the number of samples received by crop district. In this report, the production data used for each crop district to calculate the weighted quality averages are estimated production numbers since small production data are usually published by Statistics Canada [2] well after the harvest survey reports are posted. Yearly production estimates have also been difficult to assess due to a continual increase in canola production since 2000 and the large effects of the environment on the crop district production, e.g., flood in Manitoba in 2011 (Table 2).

Estimated crop district production numbers take into account the production differences between regions. The production estimations were made using five-year production averages for each crop district until 2010. In 2011, three-year production averages were chosen to lessen the discrepancy due to the steady production increase. The number of samples received in the harvest survey varied from year to year (Table 1). Production as well as quality of the crop was assumed to affect the number of samples received. Over the years, it was expected that more samples would be submitted to the harvest program if there were higher incidences of crop damage; however, Table 1 did not show a relationship between crop damage and the number of samples received.

On average for the harvest years of 2000 to 2007, one sample represented approximately 4000 tonnes. For the 2008 harvest there was an increase of over 3000 tonnes. There has been a steady rise in tonnage from 2008 to 2014. On average, 1 sample represents approximately 8266 tonnes. The highest ratio tonnes/sample was observed in 2013, which was a bumper crop year, where 1 sample represented 11,313 tonnes.

Since 2000, canola yield averages steadily increased, but that increase was not reflected equally in each province. Since 2000, on average there was a yearly yield increase of 29, 45, 57 and 22 Kg/hectare for Manitoba, Saskatchewan, Alberta and British Columbia, respectively. At the same time, Statistics Canada reported that in Canada, the number of land crop farms decreased (215,581 in 2001 versus 174,343 in 2011) while the average size of the farms increased (417 hectares in 2001 versus 501 hectares in 2011).

This could partially explain why the number of samples received in the harvest survey did not increase directly as a function of the production. It is also true that not all producers return their envelopes. The canola harvest survey shows that canola producers have between 37% and 40% envelop return rate. This highlights one of the limitations of the harvest survey program. It is voluntary and only willing producers participate; as such, the number of samples varies from year to year. As a result of the variation, the grade distribution for each crop district and the provincial averages might not be as accurate as they should be.

Export cargo data (ECD) and the number of cargoes analyzed as part of the export cargo-monitoring program from 2000 to 2014 are presented in Table 1. Overall, a little less than 50% of the Canadian canola production has been exported, and over 95% the cargoes were leaving from the port of Vancouver (data not shown). Prince Rupert, also located in British Columbia, has been marginally used to export canola cargoes.

### 2.2. Oil Content

**Figure 2:**
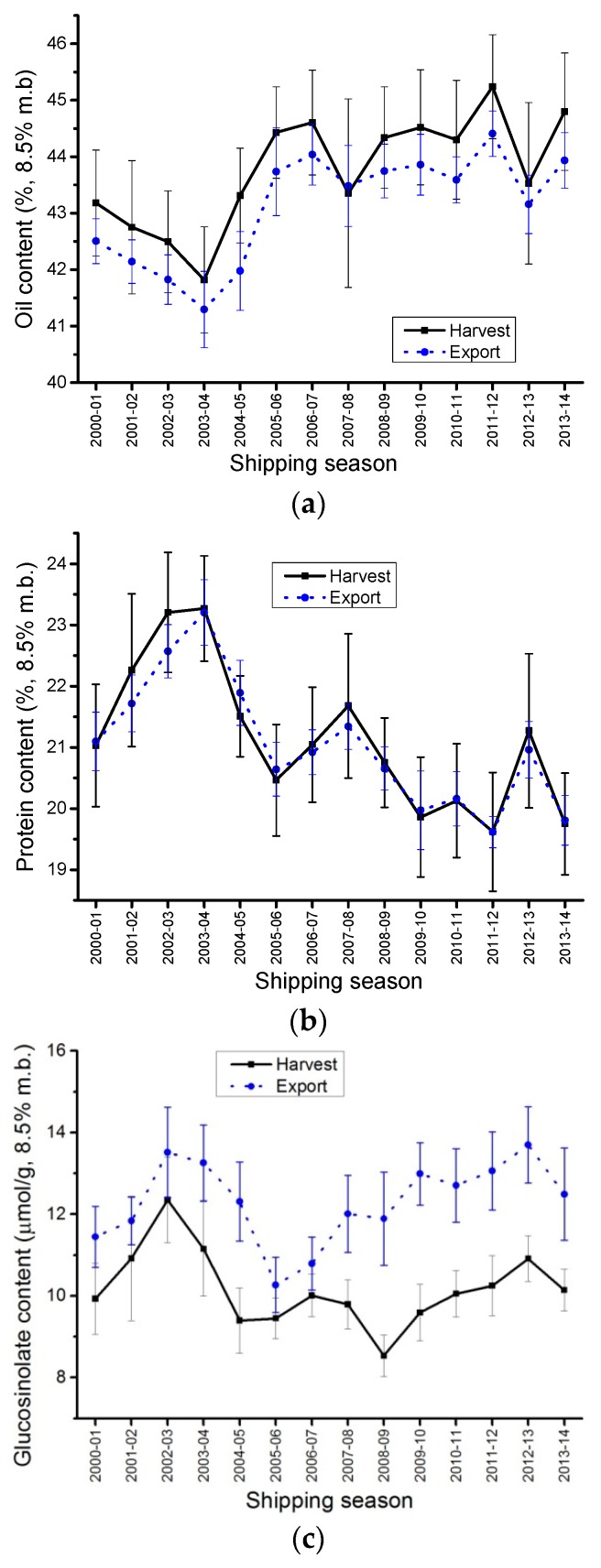
Canola oil, protein and total glucosinolate content averages (at 8.5% moisture basis), harvest (HSD) and export (ECD) samples of Canada No.1, Canola from 1 August 2000 to 31 July 2014. (**a**) Canola oil content averages (%); (**b**) Canola protein content averages (%); (**c**) Total glucosinolate content averages (µmol/g).

There was a statistical difference between the oil content of the HSD and the oil content of the ECD (Table 3, Figure 2a). There was a linear relationship between the HSD and the ECD oil contents (*R^2^* = 0.93976); the HSD oil content was always about 0.64% higher than the ECD averages. In 2005, DeClerq and Daun [3] also reported that HSD oil content averages were 0.25% higher than ECD oil content average. The harvest samples are cleaned before analyses (0.00% dockage), and they contain only inconspicuous admixture (seeds that are not readily distinguishable from canola). Canola cargo exports are considered commercially clean when they contain less than 2.5% dockage. On average, Canadian commercially-clean canola exports contained 1.88% of dockage (Table 1), whereas the harvest samples contained 0.00% dockage. Dockage is defined in the Official Grain Grading Guide as any material intermixed with canola other than canola seed [1]. It is usually made of roughage material, seed pods, knuckles or other seeds such as wheat, barley or flax and also, sometimes, broken canola seeds. Dockage material contains less oil than canola seeds, therefore, commercially clean canola samples are likely to have a lower oil content than completely clean canola samples. Every year producers grow canola seeds with higher oil content, which in turn increases the difference in oil content between the canola seeds and dockage material. The bigger difference observed in this report when compared to the 2005 report [3] was likely due to the increase in oil content of the canola seeds. It would be commercially impossible to have exports with no dockage since there is a fine balance between removing all foreign material (using various size sieves) and eliminating/losing canola seeds during the cleaning.

As a result, on average, ECD will always show lower oil content when compared to HSD. The differences in oil content between commercially clean exports and the harvest survey samples graded Canola No. 1, Canada will continue to increase as canola oil content increases with breeding.

### 2.3. Protein Content

There was no statistical difference between the protein content of the HSD and the protein content of the ECD (Table 3, Figure 2b). This agreed with DeClercq and Daun’s [3] findings. Unlike oil content, dockage had no effect on the protein content of the ECD when compared to HSD. Slight differences were observed between averages of the 2001 HSD and its corresponding ECD and between the 2002 HSD and its corresponding ECD, however, there was no statistical difference between the averages.

There were larger variations for HSD oil and protein contents than for ECD (higher standard deviation). It is known that growing conditions have important effects on oil and protein contents. The narrower variations observed in the ECD were likely due to how the grain is handled in Canada. In western Canada, producers deliver seeds to primary grain elevators where they are combined with seed from another location before moving to terminal elevators to be exported. In the primary elevators, the seeds of the same grade are combined and mixed leading to an averaging of quality and a reduction of the variations. Exported seeds also tend to be collected from geographically narrower areas than the whole western part of Canada; seeds exported via the ports in the west (Vancouver and Prince Rupert) are more likely drawn from growing areas closer to export points to reduce transportation risk and cost, for example, crop districts No. 4A and 4B from Alberta or the BC Peace River. Narrowing the growing area where the seeds are exported from will result in a narrowing of the oil and protein variation ranges.

### 2.4. Glucosinolates Content

Total glucosinolates content overall showed a statistical difference between the ECD and the HSD averages (Table 4). ECD averages were about 2 µmol/g higher than the HSD averages (Figure 2c). Again, the difference was probably due to dockage. However, it was unlikely that dockage contained components with high glucosinolates contents. Glucosinolates are very low in Canadian canola. The analytical method used to measure them might be responsible for the difference. Due to the low amount of glucosinolate, the NIR method might not be accurate as a reference method because dockage components could create artifacts leading to a slight overestimation of the glucosinolates content of the ECD compared to the HSD. However, even though a statistical difference was observed, it was a very slight. One could conclude that glucosinolates for the HSD data are able to predict glucosinolates ECD.

### 2.5. Chlorophyll Content

**Figure 3:**
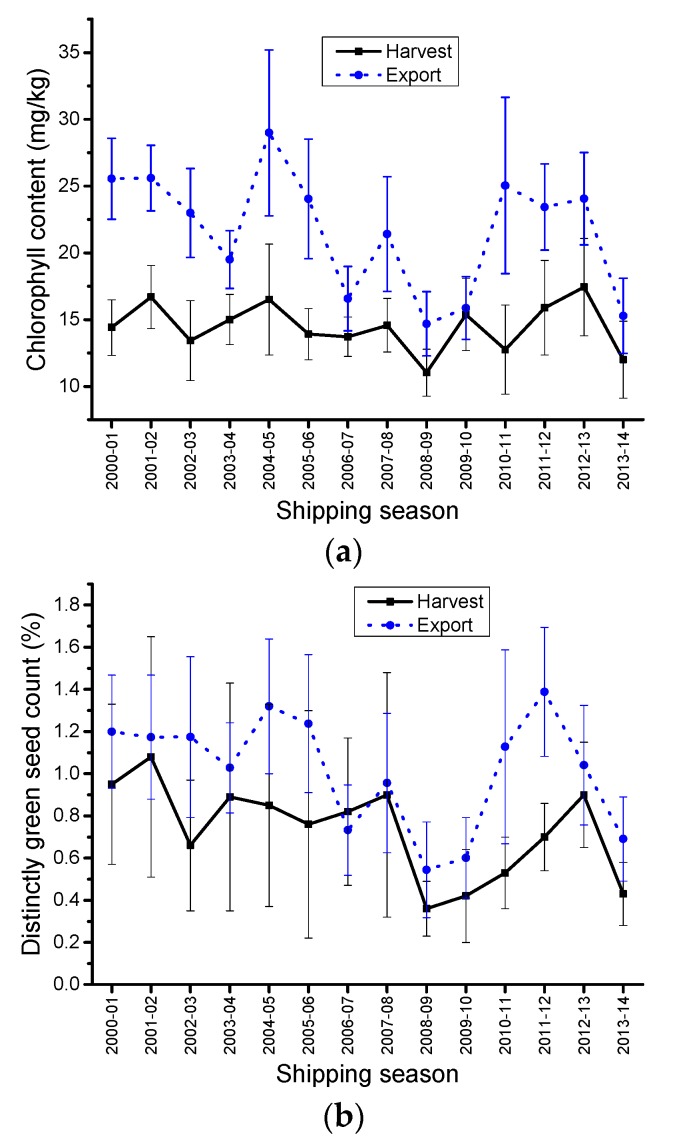
Chlorophyll content and green seed count averages, harvest (HSD) and export (ECD) samples of Canada No.1, Canola from 1 August 2000 to 31 July 2014. (**a**) Canola chlorophyll content averages; (**b**) Green seed count averages.

Large differences were observed for Chlorophyll content between HSD and ECD. On average the chlorophyll content for HSD was 7 mg/kg lower than ECD (Table 3, Figure 3a). The same 7 mg/kg difference was observed in a 2005 report [3]. The highest mean differences were observed between 2004 and 2005 ECD and its corresponding 2004 HSD (12.49 mg/kg) (Figure 3a). The lowest difference was observed between 2009 and 2010 ECD and its corresponding 2009 HSD (0.48 mg/kg) (Figure 3a). Unlike oil content, there was a very poor linear relationship between HSD and ECD chlorophyll contents (*R^2^* = 0.35979), which suggests that factors other than the amount of dockage was responsible for the mean differences. Chlorophyll is directly affected by the environment, and important geographic differences are observed almost every year in Canada, leading to geographical differences in chlorophyll content of the samples. It has also been shown that dockage quality more than quantity was affecting the chlorophyll content for the exports [4].

In 2004, the Western Prairie region experienced a very short, cold summer, with early frost in mid-August. This led to large amounts of immature and frost damaged seeds in the harvested canola. The 2004 frost damaged canola seeds were green (showing very high levels of chlorophyll), shriveled/shrunken and smaller than normal canola seeds. During grading, these frost damaged seeds became part of the dockage in the exports, whereas they were eliminated from the harvest samples during the cleaning step. The frost damaged seeds which were very high in chlorophyll content led to a significant increase in chlorophyll for the 2004–2005 ECD when compared to the HSD chlorophyll content averages of clean samples graded Canola No.1, Canada.

**Figure 4:**
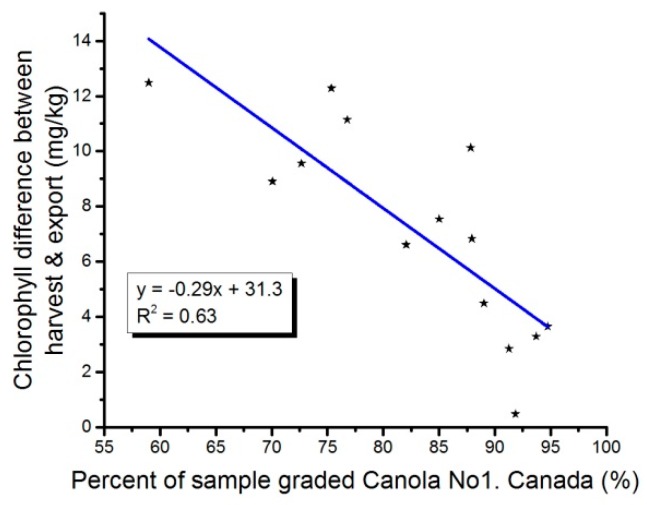
Difference between harvest and export chlorophyll averages versus the percent of harvest samples graded Canada No. 1, Canola.

Chlorophyll is not a grading factor. Distinctly Green seed (DGR) count is used for grading canola Green seed, and the tolerances are defined by the Official Grain Grading Guide [1]. Canola samples graded Canola, No.1 Canada contain a maximum of 2% DGR. There is a relationship between DGR counts and chlorophyll content. Overall, the more DGR present in a sample of canola, the higher the chlorophyll content will be. However, this is not a linear relationship [5,6]. DGR variations for HSD and ECD surveys are presented in Figure 3b. There is a statistically significant difference between ECD and HSD DGR (*p* = 0.0005). Most years, export samples show higher DGR than the harvest survey samples (Table 3). Figure 4 suggested that the amount of samples graded other than Canola No.1 Canada had an influence on the chlorophyll content differences between HSD and ECD; less Canola No.1. Canada samples resulted in a higher difference. This suggested a possible commingling of the other grades into the Canola No.1 Canada. As suggested by Daun and Symons [5], this was likely due to the green perception difference between individual grain inspectors and the sampling effect. They reported [5] that at 2% DGR using 1000 seeds for grading, the DGR could vary from 0.96% to 3.0%, 19 times out of 20. Over the 2000–2014 period, DGR has been the factor responsible of the downgrading of canola from Canola No. 1 Canada to Canola No. 2 Canada and Canola No. 3 Canada. This points out the difficulty in assessing DGR accurately; the result being the commingling of some of the lower grade canola into Canola No.1 Canada at the various step of the grain handling chain between the producers (harvest survey) and the terminal at the port (exports).

### 2.6. Free Fatty Acid Content of the Oil

**Figure 5:**
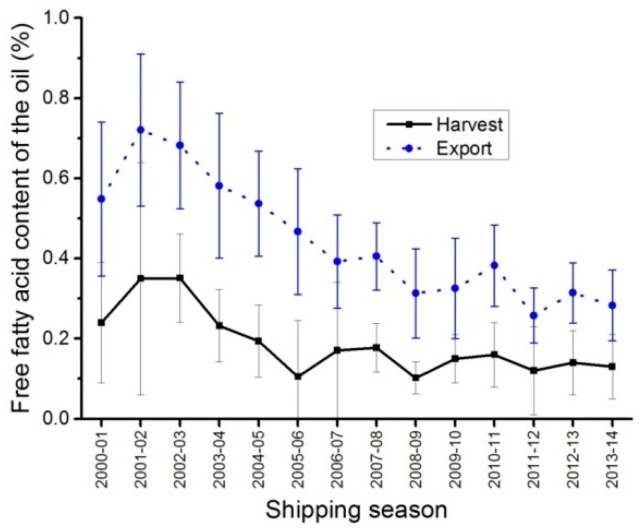
Free fatty acid content of the oil averages, harvest and export samples of Canada No. 1, Canola from 1 August 2000 to 31 July 2014.

Harvest free fatty acid averages were lower than export free fatty acid average by about 0.26% (Figure 5, Table 4). Small, shrunken/withered and broken seeds are part of dockage. These seeds are damaged leading to an increase of the free fatty acid of their oil due to break down or damage of the cellular structures. As a result, commercially clean exports with 2.5% dockage will have higher free fatty acid contents than clean samples. It has also been noted that free fatty acid content could increase during storage also leading to an increase of the export free fatty acid contents when compared to the harvest data.

### 2.7. Fatty Acid Composition of the Oil

**Figure 6:**
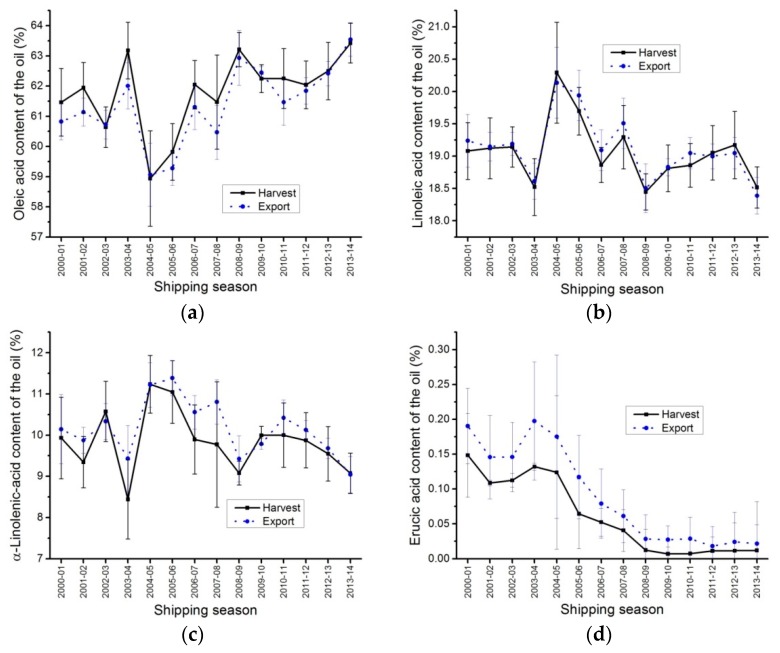

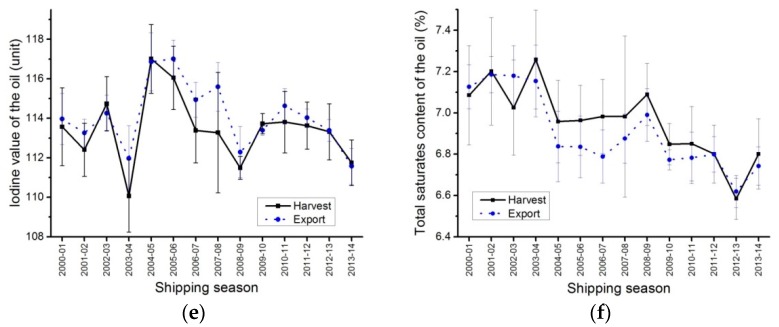
Oil quality, oleic acid, linoleic acid, α-linolenic acid, erucic acid, iodine value and total saturate content averages, harvest (HSD) and export (ECD) samples of Canada No. 1, Canola from 1 August 2000 to 31 July 2014. (**a**) oleic acid content averages (%); (**b**) linoleic acid content averages (%); (**c**) α-Linolenic acid content averages (%); (**d**) erucic acid content averages (%); (**e**) iodine value (units) averages; (**f**) total saturates content averages (%).

Oleic, linoleic, α-linolenic, erucic acid contents, total saturates and iodine value of the oil for export closely followed the harvest averages (Figure 6, Table 4 and Table 5). Statistical differences were observed between the harvest and export averages for oleic, α-linolenic and erucic acid, iodine value and total saturates (Table 4 and Table 5), however, the differences were small and had no practical effect.

Erucic acid contents have been very low since 2009; harvest and export erucic acid content averages of the oil have been well below 0.05%, with individual samples (harvest or export) showing an erucic acid range of 0.00% to 0.10% (Figure 6d and Table 5).

These data suggested that the fatty acid composition for the HSD data could be used to predict the ECD fatty acid composition.

## 3. Material and Methods

### 3.1. Harvest Data

**Figure 7:**
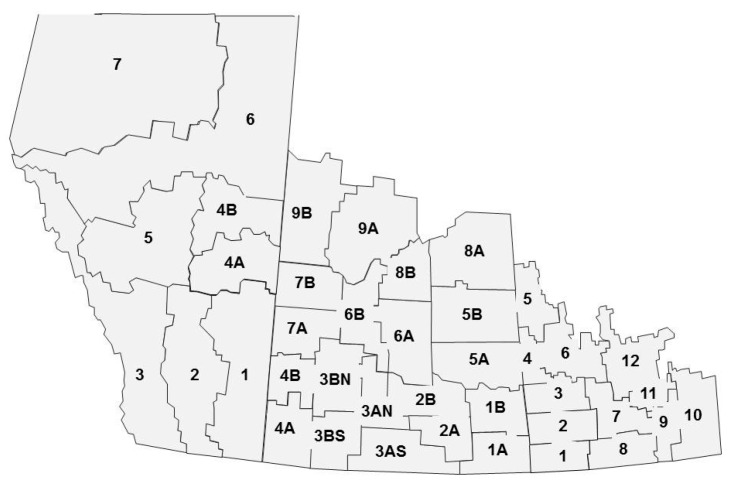
Scheme of the western provinces (Manitoba, Saskatchewan and Alberta) with the crop district divisions used in the Canadian Grain Commission harvest survey.

The harvest data are from the Canadian Grain Commission’s annual harvest program. Quality data are from analyses of canola samples submitted to the Canadian Grain Commission throughout the harvest period by producers, grain companies and oilseed crushing companies. The data on quality for the reported years were based on the analyses of the number of samples received in the harvest survey and the number of samples graded Canola No. 1 Canada (N_H1_) (Table 1). Composites of Canola No. 1 Canada from the various crop districts for each province are made yearly to be analyzed by reference methods. The reported harvest data included only conventional canola samples (no specialty oil samples). Provincial and western Canadian averages were calculated from results for each crop district (Figure 7), weighted by a combination of production by crop district (Figure 1) using Statistics Canada production estimates [2], combined with an estimate of grade distribution per crop district using the Canadian Grain Commission data (Figure 7). Each sample submitted to the harvest sample program is officially graded by inspectors of the Industry Services Division of the Canadian Grain Commission using the Official Grain Grading Guide for canola and rapeseed [1]. Harvest samples are collected from 1 August to 1 December of the same calendar year. Harvest data from 2000 to 2014 were used in this report.

### 3.2. Export Data

Exports of commercially cleaned canola exports (from 1 August to 31 July of the following calendar year) were used in the report. Canola exports containing less than 2.5% dockage are considered commercially clean. The export quality average was calculated using each cargo quality data weighted with the cargo tonnage. Export data from 2000 to 31 July 2014 were used in this report.

### 3.3. Methods

Quality parameters used are oil, protein, chlorophyll, glucosinolates, free fatty acids and the fatty acid composition. Oil, protein and glucosinolates content values are presented using the Canadian Grain Commission’s historical 8.5% moisture basis. Protein content was determined using the AOCS Official method [7]; results are reported as a percentage using N × 6.25. Oil content, expressed as %, was determined by nuclear magnetic resonance (NMR) according to the International Organization for Standardization [8]. The NMR instrument was calibrated with oilseed samples analyzed with the ISO reference method [9]. Glucosinolate content was determined by NIR calibrated with the total glucosinolate method [10]. Chlorophyll content was determined spectrometricaly [11] and results were expressed as milligrams per kilogram (mg/kg), seed basis. Fatty acid composition was determined after base catalyzed derivatization [12] using a gas chromatograph equipped with a FID detector [13]. The results are presented as a relative fatty acid composition. Iodine value which is a measure of unsaturation was calculated from the fatty acid composition according to AOCS recommended practice [14]. The free fatty acid content of the oil was determined using Ke et al.’s method [15] and the results are expressed as a percentage by weight of oleic acid in the oil.

### 3.4. Statistic and Analysis

The harvest quality averages for the year (1 August to 1 December of the same calendar year) were compared with the export quality averages of the corresponding shipping season (1 August of the year of harvest to 31 July of the following calendar year). Analyses were done using Instat version 3.10 (GraphPad, San Diego, CA, USA). Paired t-tests with two tail *p*-value were used to compare the yearly averages. Origin version 9.1.0 (Origin Lab Corporation) was used to produce the graphs.

## 4. Conclusions

Canola harvest survey data can be extrapolated to predict the quality of the Canadian Canola exports. Some differences were constant, e.g., Canadian exports contained lower oil content when compared to the oil content of the Harvest Survey and the difference was mainly due to dockage, which remained constant over the years (1.7% to 1.9%). Chlorophyll was the least predictable of all the parameters; dockage quality, as well as commingling of the other grades in Canola No. 1 Canada affected the chlorophyll content of the exports and those effects are difficult to predict from one cargo to another or one year to another. Other parameters such as protein content and fatty acid composition were very similar for harvest and export averages. Free fatty acids also showed some differences, however, they are affected by storage and as such are difficult to predict.

The results of this study suggested that the canola data quality obtained by the CGC harvest survey program could be used to forecast the quality of the canola exported by shipments for the corresponding season. Canola harvest survey data are a reliable descriptor of the yearly quality of western Canadian canola and could be used as a tool to support the marketing of Canadian canola.

## Figures and Tables

**Table 1:** Summary table—Canadian canola production, number of samples received in the harvest survey with the percent of samples graded Canada No. 1 Canola, number of export cargoes analyzed by the monitoring program and the tonnage they represent (average, minimum and maximum), and percent dockage average of the export cargoes from 1 August 2000 to 31 July 2014.

Year of Harvest	Shipping Season	Canada Production (Tonnes) *	Number of Samples in Harvest Survey (N_H_)	Percent of Canola, No.1 Canada (N_H1_)	Number of Cargoes (N_E_)	Total Export (Tonnes)	Export Average (Tonnes)	Minimum Export Tonnage (Tonnes)	Maximum Export Tonnage (Tonnes)	Dockage (%)
2000	2000–01	7,205,300	1386	76.77	148	4,180,554	28,246.99	498	57,750	1.97 ± 0.21
2001	2001–02	5,017,100	1343	70.07	97	2,244,769	23,141.93	848	52,376	1.96 ± 0.30
2002	2002–03	4,520,500	1266	72.67	83	1,683,828	20.287.08	4200	51,569	1.90 ± 0.27
2003	2003–04	6,771,200	2203	89.01	114	2,695,243	23,642.48	2100	56,500	1.88 ± 0.21
2004	2004–05	7,673,600	1943	58.98	100	2,240,216	22,402.16	3489	55,002	1.80 ± 0.25
2005	2005–06	9,483,300	2186	87.83	158	4,270,430	27,028.04	2000	60,500	1.69 ± 0.38
2006	2006–07	9,000,300	2485	91.27	149	3,880,916	26,046.42	3290	60,500	1.86 ± 0.27
2007	2007–08	9,611,300	2114	87.94	151	3,609,326	23,902.82	2017	65,253	1.94 ± 0.20
2008	2008–09	12,644,900	1767	94.74	196	6,351,669	32,406.47	3833	65,879	1.77 ± 0.29
2009	2009–10	12,898,100	1362	91.85	183	5,678,678	31,031.03	3249	65,556	1.91 ± 0.35
2010	2010–11	12,788,600	1785	75.35	169	5,161,053	30,538.78	1900	63,225	1.88 ± 0.28
2011	2011–12	14,608,100	1749	85.02	193	6,465,592	33,500.48	3427	69,550	1.89 ± 0.27
2012	2012–13	13,868,500	2089	82.05	168	5,080,798	30,242.85	1236	63,800	1.99 ± 0.27
2013	2013–14	17,965,800	1588	93.70	192	7,190,048	37,448.17	5250	66,000	1.92 ± 0.40

* Data published by Statistics Canada.

**Table 2:** Crop district summary table—Canola production per crop district (average, minimum and maximum) and number of samples received by the annual harvest survey program (average, minimum and maximum) from 1 August 2000 to 31 July 2014.

		Production (Tonnes)			Number of Samples in Harvest Survey		
Census Ag Region	Canola Crop District	Mean	Min.	Max.	Mean	Min.	Max.
Manitoba							
1	1	206,779	72,478	376,485	46	11	74
2	2	193,470	119,699	321,300	54	21	86
3	3	231,997	136,492	450,600	57	36	85
4	4	95,743	51,346	212,900	35	23	52
5	5	156,529	94,986	202,250	27	17	40
6	6	137,766	87,162	228,942	38	21	54
7	7	257,834	100,567	436,800	61	9	95
8	8	360,678	192,968	560,406	95	49	139
9 + 10	9 + 10	92,842	15,313	145,012	38	14	62
11	11	65,398	19,136	102,257	16	6	31
12	12	55,403	23,374	86,500	10	3	17
Manitoba		1,854,443	1,122,600	2,871,200	476	332	662
Saskatchewan							
11 + 12	1	389,742	120,331	902,800	72	20	111
21 + 22	2	295,099	85,754	878,400	71	41	94
31 + 32 + 33 + 34	3	204,586	55,722	658,700	45	17	80
41 + 42	4	34,209	4064	114,500	9	3	15
51 + 52	5	911,496	495,214	1,696,200	172	129	231
61 + 62	6	729,810	221,867	1,316,600	141	94	203
71 + 72	7	376,404	38,217	918,600	79	22	115
81 + 82	8	752,037	209,092	1,390,130	127	75	184
91 + 92	9	723,526	81,082	1,245,800	119	45	158
Saskatchewan		4,414,900	1,769,000	8,917,600	835	571	1085
Alberta + British Columbia							
10	1	85,002	17,189	218,134	12	4	19
20	2	540,415	116,814	1,154,993	81	37	117
30	3 + 1 *	229,430	37,760	546,300	44	12	67
40	4A + 4B	938,368	133,001	1,717,600	193	62	262
50	5	492,696	164,888	771,400	62	32	97
60	6	342,346	148,350	590,500	33	21	46
70	7 + 8 **	694,917	452,849	1,188,600	91	50	130
AB + BC		3,470,600	1,242,800	6,087,500	517	274	677
Canada		9,739,943	4,463,300	17,876,300	1827	1300	2337

* Samples from crop district #1 from British Columbia were combined with samples from Alberta crop district #3; ** Samples from crop district #8 from British Columbia were combined with samples from Alberta Crop district #7.

**Table 3:** Yearly mean average and standard deviation for harvest (HSD) and exports (ECD) samples of Canada No. 1, Canola for green seed count, oil, protein and chlorophyll content.

	Distinctly Green Seeds (%)		Oil Content (%, 8.5% m.b.)		Protein Content (%, 8.5% m.b.)		Chlorophyll Content (mg/kg)	
	Harvest	Export	Harvest	Export	Harvest	Export	Harvest	Export
Average	0.73	1.02	43.76	43.12	21.13	21.04	14.48	21.64
Standard deviation	0.22	0.27	0.99	0.98	1.18	1.04	1.84	4.52
Minimum	0.36	0.54	41.82	41.30	19.62	19.61	11.03	14.68
Maximum	1.08	1.39	45.24	44.41	23.27	23.20	17.43	28.99
Median								
Paired *t*-test—Two tail *p* value—Results								
Mean difference	−0.28540		0.64000		0.09286		−7.16300	
*p* value	0.0005		0.00010		0.23670		<0.0001	
*t*	4.322		7.674		1.240		7.078	

**Table 4:** Yearly mean average and standard deviation for harvest (HSD) and exports (ECD) samples of Canada No. 1, Canola for free fatty acid, total glucosinolates, and total saturates contents.

	Total Glucosinolates Content of the Seeds (µmol/g)		Free Fatty Acid Content of the Oil (%)		Iodine Value of the Oil (Units)		Total Saturates Content of the Oil (%)	
	Harvest	Exports	Harvest	Export	Harvest	Export	Harvest	Exports
Average	10.18	12.31	0.19	0.44	113.45	114.08	6.96	6.91
Standard deviation	0.93	1.01	0.08	0.15	1.77	1.65	0.18	0.19
Minimum	8.53	10.27	0.10	0.26	110.07	111.57	6.58	6.62
Maximum	12..35	13.7	0.35	0.72	117.01	117.01	7.26	7.18
Median	10.03	12.4	0.16	0.40	113.48	114.00	6.97	6.84
Paired *t*-test—Two tail *p* value Results								
Mean difference	−2.13		−0.2564		−0.6371		0.05286	
*p* value	<0.0001		<0.0001		0.0150		0.0410	
*t*	8.551		11.336		2.802		2.268	

**Table 5:** Yearly mean average and standard deviation for harvest (HSD) and exports (ECD) of samples of Canada No. 1, Canola for oleic, linoleic, α-linolenic and erucic acid contents of the oil.

	Oleic Acid Content of the Oil (C18:1, %)		Linoleic Acid Content of the Oil (C18:2, %)		α-Linolenic Acid Content of the Oil (C18:3, %)		Erucic Acid Content of the Oil (C22:1, %)	
	Harvest	Export	Harvest	Export	Harvest	Export	Harvest	Export
Average	61.80	61.39	19.06	19.12	9.84	10.16	0.06	0.09
Standard deviation	1.28	1.28	0.49	0.49	0.76	0.68	0.05	0.07
Minimum	58.94	59.07	18.45	18.39	8.44	9.04	0.01	0.02
Maximum	63.43	63.54	20.29	20.14	11.24	11.38	0.15	0.20
Median	62.04	61.38	19.07	19.07	9.88	10.14	0.04	0.07
Paired *t*-test—Two tail *p* value—Results								
Mean difference	0.4093		−0.05857		−0.3157		−0.03214	
*p* value	0.0054		0.1300		0.0104		<0.0001	
*t*	3.334		1.616		2.993		5.993	
